# Genetic and environmental influences on fruit and vegetable consumption and depression in older adults

**DOI:** 10.1186/s12877-023-03745-0

**Published:** 2023-02-03

**Authors:** Annabel P. Matison, Anbupalam Thalamuthu, Victoria M. Flood, Julian N. Trollor, Vibeke S. Catts, Margaret J. Wright, David Ames, Henry Brodaty, Perminder S. Sachdev, Simone Reppermund, Karen A. Mather

**Affiliations:** 1 Centre for Healthy Brain Ageing, Discipline of Psychiatry and Mental Health, Faculty of Medicine and Health, UNSW Sydney, Level 1, AGSM (G27) Gate 11, Botany Street, Sydney, NSW 2052 Australia; 2 Faculty of Medicine and Health, The University of Sydney, Sydney, NSW Australia; 3 University Centre for Rural Health, Northern Rivers, Lismore, NSW Australia; 4 Department of Developmental Disability Neuropsychiatry (3DN), Discipline of Psychiatry and Mental Health, Faculty of Medicine and Health, UNSW Sydney, Sydney, NSW Australia; 5 Queensland Brain Institute, University of Queensland, Brisbane, QLD Australia; 6 Centre for Advanced Imaging, University of Queensland, Brisbane, QLD Australia; 7 Academic Unit for Psychiatry of Old Age, University of Melbourne, St George’s Hospital, Melbourne, VIC Australia; 8 National Ageing Research Institute, Melbourne, VIC Australia; 9 Neuropsychiatric Institute, Prince of Wales Hospital, Sydney, NSW Australia

**Keywords:** Fruit, Vegetables, Depression, Genetics, Heritability, Twin study

## Abstract

**Background:**

Prior work suggests that higher fruit and vegetable consumption may protect against depression in older adults. Better understanding of the influence of genetic and environmental factors on fruit and vegetable intakes may lead to the design of more effective dietary strategies to increase intakes. In turn this may reduce the occurrence of depression in older adults.

**Objectives:**

The primary aim of this study is to estimate the genetic and environmental influences on the consumption of fruit and vegetables in older adults. The secondary aim is an exploratory analysis into possible shared genetic influences on fruit and vegetable intakes and depression.

**Methods:**

Analysis of observational data from 374 twins (67.1% female; 208 monozygotic (MZ); 166 dizygotic (DZ)) aged ≥ 65 years drawn from the Older Australian Twins Study. Dietary data were obtained using a validated food frequency questionnaire and depressive symptoms were measured using the 15-item short form Geriatric Depression Scale. The contribution of genetic and environmental influences on fruit and vegetable intake were estimated by comparing MZ and DZ twin intakes using structural equation modelling. A tri-variate twin model was used to estimate the genetic and environmental correlation between total fruit and vegetable intakes and depression.

**Results:**

In this study, vegetable intake was moderately influenced by genetics (0.39 95%CI 0.22, 0.54). Heritability was highest for brassica vegetables (0.40 95%CI 0.24, 0.54). Overall fruit intake was not significantly heritable. No significant genetic correlations were detected between fruit and vegetable intake and depressive symptoms.

**Conclusions:**

Vegetable consumption, particularly bitter tasting brassica vegetables, was significantly influenced by genetics, although environmental influences were also apparent. Consumption of fruit was only influenced by the environment, with no genetic influence detected, suggesting strategies targeting the food environment may be particularly effective for encouraging fruit consumption.

**Supplementary Information:**

The online version contains supplementary material available at 10.1186/s12877-023-03745-0.

## Background

Depression is a common mental health condition in older adults and is associated with reduced quality of life [1]. Estimates of the global prevalence of depression vary considerably depending on the population examined and the definition of depression used; however, estimates of major depression in older adults are generally in the range of 1% to 3% [2]. Further contributing to this prevalence, it is estimated that approximately 10% of older adults experience depressive symptoms, which do not reach the threshold of a clinical depression diagnosis but still adversely impact health-related quality of life [3, 4].

Some evidence suggests that diet is associated with depression, both in the general population and in older adults. Our own meta-analysis of longitudinal observational studies in adults ≥ 45 years (4 studies, total participants 176,649) found higher baseline fruit and vegetable intakes were associated with reduced risk of developing depression during the follow-up period (15% and 9% reduction respectively) [5]. Studies in the general population suggest that adopting a healthy diet, for example one high in fruits and vegetables, may reduce depressive symptoms [6].

Despite higher fruit and vegetable intake being associated with numerous physical health benefits (including reduced risk of cardiovascular disease, total cancer and all-cause mortality) [7] and emerging evidence suggesting mental health benefits [8], few older adults meet dietary guidelines for fruit and vegetable intake [9]. Over 37% of Australians aged ≥ 65 years do not consume the recommended daily fruit intake, with this figure rising to 89% for vegetables [10]. Low fruit and vegetable intake is also an issue in other Western countries [11, 12]. New strategies are required to increase these intakes. Prior work has established that genes and the environment influence both dietary intake [13] and depression [14] and these influences appear to vary by age.

The classic twin model can be used to estimate how much of the variation in a trait (in this case fruit and vegetable intake and depressive symptoms) between twins is due to genetic and environmental influences. Using the difference in genetic relatedness between MZ and DZ twins, the variance in traits between twins can be attributed to either genetic or environmental influences, with the latter further attributed to either their shared environment (e.g. living in the same household as children) or their unique environment (e.g. living in different households as adults). The proportion of variation in trait attributed to genetics is referred to as the “heritability” of the trait.

Little is known about the heritability of diet in adults aged ≥ 65 years. Most studies into the heritability of diet have used a general adult population [15–17] or youth [18, 19]. Only one study has examined an older population, with moderate heritability of dietary intake of both a “healthy” and “unhealthy” diet reported in a US cohort of twins aged ≥ 50 years [20]. Additionally, it appears that intakes of some fruit and vegetables are more heritable than others. For example, in a UK cohort of twins aged ≥ 18 years the heritability of garlic intake was estimated to be 46% while intake of fruit juice appeared not to be heritable [16]. One explanation for these differences in heritability is genetically driven differing taste perceptions [21]. Another question of interest is whether any association between higher fruit and vegetable intake and reduced symptoms of depression is due to underlying genetic factors. It has previously been reported that macronutrient intakes and depression do not share a genetic (or phenotypic) correlation [22], but this question appears not to have been examined for fruit and vegetable intakes. A genetic correlation would suggest that the same genes influencing fruit and vegetable intake are also important to the risk of depression.

By better understanding the influence of genetic and environmental factors on dietary intakes of foods that are important to the risk of depression, more effective dietary strategies could be designed to reduce and/or assist with the treatment of depression in older adults. The primary aim of this study was to estimate the genetic and environmental influences on the consumption of fruits and vegetables in older adults. The secondary aim was an exploratory analysis into whether there are shared genetic influences on fruit and vegetable intakes and depression.

## Methods

### Study design and population

This study uses data from the Older Australian Twins Study (OATS), a longitudinal observational study which commenced in 2006 and was designed to investigate the genetic and environmental factors associated with healthy brain ageing. OATS recruited 623 twins and their siblings, aged 65 years and older and residing in New South Wales, Victoria or Queensland from the Australian Twin Registry. Inclusion criteria involved having an ability to consent, a co-twin willing to consent and having adequate English to complete a neuropsychological assessment. Participants were excluded if they had a life-threatening illness, a current diagnosis of malignancy or an acute psychotic disorder. For further details see Sachdev et al. [23].

**Fig. 1:**
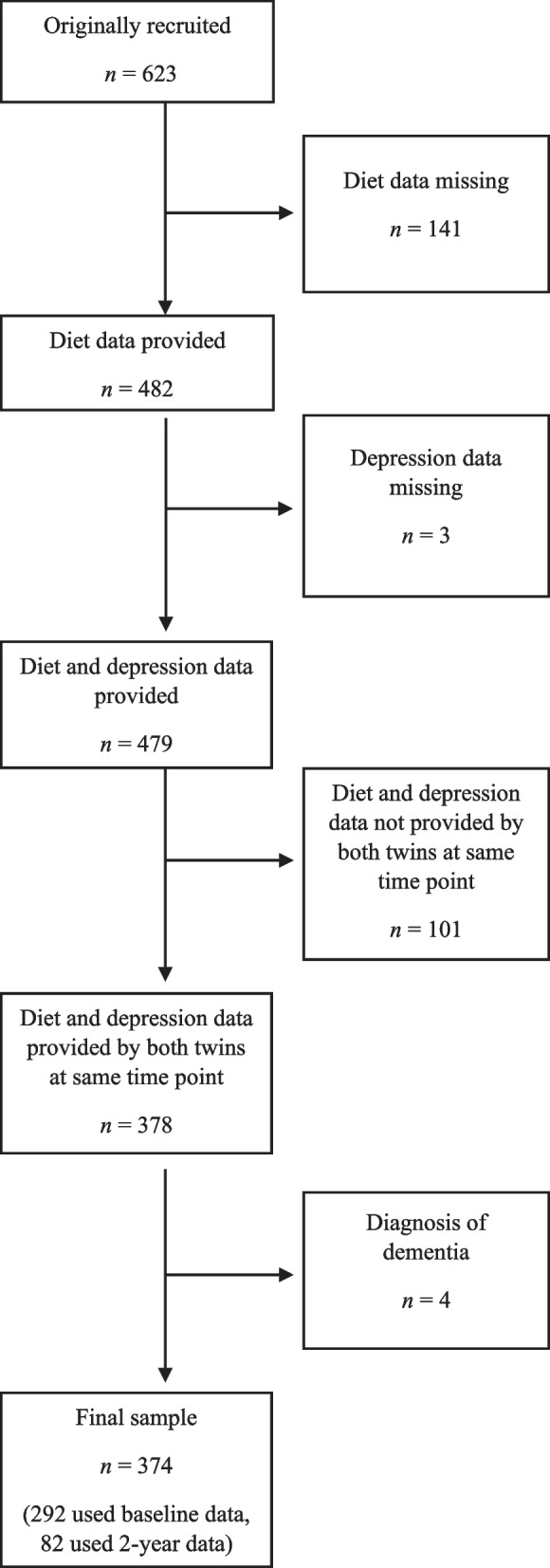
Flowchart of participants and selection process

The current study uses data collected at one of two time points (either baseline or approximately two years later) and is restricted to the 374 twins (MZ: 208; DZ: 166) who were free from dementia and provided a valid dietary and depression assessment at the same time point either at baseline or two years later. Where both twins did not provide the required data at the same time point, they were excluded from the study to avoid differences due to different collection time points. Figure 1 provides details of reasons participants were excluded from the final sample used for analysis, with the primary reasons being not completing a dietary assessment, or not having a co-twin completing a dietary and depression assessment at the same time point. The zygosity of the sample was assessed based on identity by descent using available genome-wide genotyping data along with self-report data [24].

### Measurements

#### Fruit and vegetable intake

Dietary intakes were obtained at baseline and 2 years later using the Dietary Questionnaire for Epidemiological Studies Version 2 (DQESv2). The DQESv2 is a 74-item food frequency questionnaire (FFQ) which is commonly used in epidemiological studies to measure “habitual” dietary intake and has been validated using weighed food records [25–27]. Data for fruit and vegetable intakes were determined using the following questions “How many pieces of fresh fruit do you usually eat per day? (count ½ cup of diced fruit, berries or grapes as one piece)” and “How many different vegetables do you usually eat per day? (count all types, fresh, frozen or tinned)”. Three different sample serving sizes were depicted and participants were asked to indicate the quantity they usually ate. Participants were then asked how often over the preceding 12 months they had consumed a list of 13 fruits and 22 vegetables (see Table 1). There were 10 response categories ranging from “never” to “3 or more times per day”. Based on the responses to these questions, nutrient composition data for Australian foods from the NUTTAB95 database [28] and using published guidelines [25], fruit and vegetable intakes in grams/day and energy intake in kilojoules/day were calculated. Where a participant did not provide a valid fruit, vegetable or energy intake, the dietary assessment was treated as invalid. Fruit and vegetable intakes were then converted to servings using serving sizes from the Australian Dietary Guidelines [29].

**Table 1:** Details of fruit and vegetable categories

Category of fruit/vegetable	Fruits and vegetables included
Fruit	Tinned fruit, juice, oranges, apples, pears, bananas, melons, pineapple, strawberries, apricots, peaches, mango, avocados
Vegetables	Potatoes (excluding roast/fried/hot chips), tomatoes (excluding tomato sauce/tomato paste/dried tomatoes), capsicum, lettuce, cucumber, celery, beetroot, carrots, cabbage, cauliflower, broccoli, spinach, peas, green beans, bean sprouts, baked beans, tofu, other beans, pumpkin, onion, garlic, mushrooms, zucchini
Tropical/citrus fruit	Oranges, bananas, melons, pineapple, mango, avocados
Stone fruit	Apricots, peaches
Other fruit	Apples, pears, strawberries
Brassica vegetables	Cabbage, cauliflower, broccoli
Root vegetables	Carrots, beetroot
Salad vegetables	Tomatoes, lettuce, cucumber, celery
Starchy vegetables	Potatoes, pumpkin
Peas/beans/legumes	Peas, green beans, bean sprouts, baked beans, tofu, other beans
Other vegetables	Mushrooms, zucchini, spinach, capsicum

#### Depressive symptoms

Depressive symptoms were self-reported at baseline and 2 years later using the 15-item short form of the Geriatric Depression Scale (GDS) [30]. The 15-item GDS has been validated for use in older adults [30, 31]. The version used in OATS included Question 9 as described by Brink [32]. A summary score ranging from 0 to 15 (where higher scores indicate greater symptoms of depression) was calculated based on participant responses and used in analyses. Where a participant did not provide a response to a question, the summary score was calculated using a pro-rata of the responses provided. If less than 80% of questions were answered the assessment was considered invalid.

#### Other variables

Participant demographics, including age, sex, years of education and living arrangement (either “in the community alone”, “in the community with spouse”, “in the community with other” or “other” which combined “in a hostel villa”, “in a retirement home”, “other” and “unknown”) were collected via questionnaire at each time point. Height and weight were collected via physical examination. Body mass index (BMI) was calculated as weight in kilograms divided by height in metres squared. Activities of daily living (ADL) score was collected using the Bayer ADL scale [33].

### Statistical analysis

Participant characteristics were presented as mean and standard deviation (SD) for continuous data and number and percentage for categorical data. Differences between MZ and DZ twins were examined using independent sample t-tests for continuous variables and chi-squared tests for categorical variables. *P*-values were obtained by permutation tests to account for related twins within the sample. Zygosity of pairs was first permutated and observations from two sets of pairs were then interchanged.

#### Genetic and environmental influence

The classic twin model [34] was used to estimate what proportion of the variation in fruit and vegetable intake and depressive symptoms was due to additive genetic influences (A), shared environmental influences (C), and unique environmental influences (E). Shared environmental influences for older adults include experiences twins have similarly experienced having been reared together (e.g. living in the same household and neighbourhood as children). Unique environmental influences are individual specific influences which the twins do not share. These are likely to include living in different households and different neighbourhoods as adults. The twin model compares the similarity of traits between MZ twin pairs and DZ twin pairs. A, C and E are estimated using the difference in relatedness between MZ twins (who share 100% genetic material) and DZ twins (who share ≈ 50% genetic material). If the intraclass correlation for a particular trait between MZ twins (MZICC) is greater than the correlation between DZ twins (DZICC), it suggests that the trait is influenced by genetic factors. If the correlation between DZ twins is greater than 50% of the correlation between MZ twins, then the trait is more similar than would be expected from shared genetics, which suggests it is influenced by shared environmental factors (C). The variation in trait between MZ twins reflects unique environment factors (and includes measurement error) (E). Twin behavioural genetics structural equation modelling (SEM) was used to estimate the relevant parameters.

Sex differences were examined using a heterogeneity model. Under this model, different sets of parameters are used for male, female and opposite sex twin pairs. The full likelihood is the sum of the separate components of these subgroups of samples. The homogeneity of the parameter estimates is tested by comparison with the model constrained by the same set of parameters for male and female samples.

#### Genetic correlation

The tri-variate twin model was used to perform our exploratory analysis into whether there are shared genetic influences on fruit and vegetable intake and depression. The tri-variate model estimated the influences of A, C and E on total fruit and vegetable intakes and depressive symptoms, and what proportion of the association between total fruit and/or vegetable intake and depressive symptoms was due to A, C and E.

All the analyses were done based on standardised residuals. For dietary variables, residuals adjusting for age, sex and energy intake were obtained using a linear regression model. For depressive symptoms, age and sex were used to obtain the residuals.

To determine the robustness of our main analysis we performed sensitivity analysis by adjusting for key variables which we considered may be on the causal pathway between genetics and fruit/vegetable intake. For example, we considered that genetics may influence a person’s education attainment [35], which in turn may influence their fruit and vegetable intake [36]. The key variables adjusted were education and BMI.

There was no missing covariate data for the main analysis. Sensitivity analysis used data with 9 missing BMI values, these were replaced by the mean BMI of the participants who did provide BMI data.

For both univariate and tri-variate SEM, the Cholesky model with the three latent variables A, C and E (ACE) was fit first and then compared against the restricted model with A and E components (AE). Goodness of fit were examined using Akaike information criteria (AIC) and the *p*-values comparing the likelihoods of ACE vs AE models. All the analyses were done using the R (4.0.0) package [37]. Univariate and tri-variate SEM for heritability and genetic correlations were performed using the R statistical package OpenMx version 2.18.1 [38]. All the confidence intervals were obtained based on 1000 bootstrap samples.

## Results

Characteristics of participants by zygosity are set out in Table 2. The mean age of the 374 participants was 70.8 ± 5.5 years (range 65 – 90 years) and 67.1% were female. The mean BMI was 27.5 ± 4.4 kg/m^2^ (mean BMI for Australians aged ≥ 65 years is 29.0 kg/m^2^ [39]). Participants appeared to be high functioning with a mean ADL score of 1.35 ± 0.47 [33]. The majority of participants were living in the community with a spouse. Mean intake of fruits and vegetables were 2.1 ± 1.4 servings/day and 2.5 ± 1.1 servings/day respectively. Only 46.5% and 2.9% of participants consumed at or above the Australian Dietary Guideline recommendation for fruit (2 serves) and vegetables (5 serves) [29] respectively. Depressive symptoms were generally low in both groups (GDS range 0 – 15). The prevalence of depression was estimated to be 7% (based on a GDS depression cut-off score of ≥ 5 [40]). The only significant difference between MZ and DZ twins was that DZ twins had a higher BMI.

**Table 2:** Characteristics of participants by zygosity

	**Total**	**MZ**	**DZ**	
	**(*n* = 374)**	**(*n* = 208)**	**(*n* = 166)**	***p-value***
Age (years), mean (SD)	70.8 (5.5)	71.0 (5.7)	70.7 (5.2)	0.662
Sex, n (% female)	251 (67.1)	140 (67.3)	111 (66.9)	0.916
BMI (kg/m^2^), mean (SD)^a^	27.5 (4.4)	27.0 (4.3)	28.1 (4.3)	0.014
Education (years), mean (SD)^a^	11.5 (3.3)	11.2 (3.3)	11.8 (3.4)	0.107
Activities of daily living score, mean (SD)^b^	1.35 (0.47)	1.34 (0.46)	1.35 (0.48)	0.941
Living arrangement^b^				
- In the community alone, n (%)	99 (26.4)	52 (25.0)	47 (28.3)	
- In the community with spouse, n (%)	231 (61.8)	128 (61.5)	103 (62.1)	0.483
- In the community with other, n (%)	34 (9.1)	23 (11.1)	11 (6.6)	
- Other, n (%)	10 (2.7)	5 (2.4)	5 (3.0)	
Energy intake, (kJ/day), mean (SD)	7121 (2683)	6881 (2661)	7422 (2689)	0.053
Fruit intake (serves/day), mean (SD)	2.13 (1.35)	2.15 (1.33)	2.12 (1.40)	0.819
Vegetable intake (serves/day), mean (SD)	2.53 (1.11)	2.52 (1.14)	2.56 (1.08)	0.739
Geriatric depression scale score, mean (SD)	1.74 (2.04)	1.66 (1.96)	1.84 (2.14)	0.415

*P-value* based on independent sample t-test for continuous variables and chi-squared test for categorical variables and using permutation tests to account for related twins within the sample. All the *p-values* were obtained based on 5000 permutations

*MZ* monozygotic, *DZ* dizygotic, *SD* standard deviation, *n* number, *%* percentage, *BMI* body mass index, *kg* kilogram, *m* metre, *kJ* kilojoule

^a^variable adjusted for in sensitivity analysis, mean BMI based on *n* = 365

^b^variable included for information only, not treated as a covariate, mean for Activities of daily living score based on *n* = 311

### Heritability analysis for the total sample

**Fig. 2:**
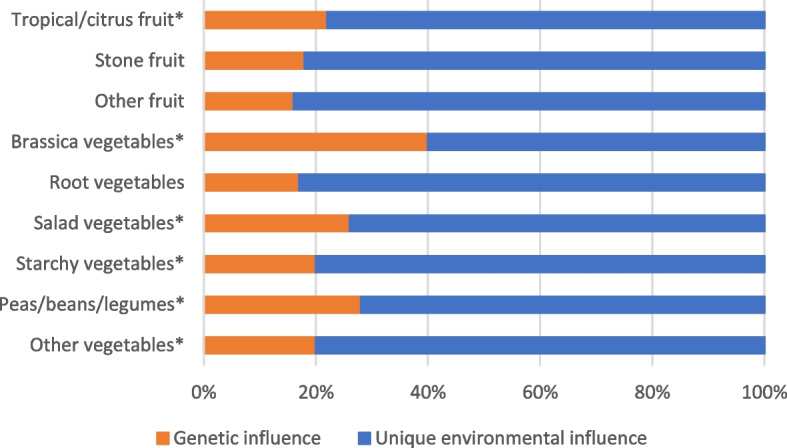
Estimate of proportion of the variation in intake by type of fruit/vegetable due to genetic and unique environmental influences under univariate AE model (*n* = 374, 104 MZ pairs, 83 DZ pairs). All intakes adjusted for age, sex and total energy intake *Heritability influence *p-value* < 0.05

The best-fitting heritability model was AE, suggesting the shared environment did not significantly influence fruit and vegetable intakes or depressive symptoms (model comparisons are included in Supplemental Table 2). Therefore, all parameters were estimated under the AE model. Results of the heritability analysis are set out in Table 3. We estimate that 39% of the variability in vegetable intake was attributable to genetics with the remaining 61% of variability due to the unique environment, while for depressive symptoms the genetic influence was 30% and unique environment 70%. There was no significant influence of genetics on the overall intake of fruit. Looking at different types of fruits and vegetables, heritability was highest for brassica vegetables and peas/beans/legumes (40% and 28% respectively), while there was no statistically significant heritability association for root vegetables (Fig. 2 and Supplemental Table 1). Tropical/citrus fruit was the only category of fruit intake with significant heritability. Heritability estimates were similar between multivariate and univariate models. Results of sensitivity analysis adjusting for education and BMI were largely unchanged from the main analysis (results included in Supplemental Table 3 and model comparisons are included in Supplemental Table 4).

**Table 3:** Intraclass correlations and heritability estimates for fruit and vegetable intakes and depressive symptoms

	**ICC MZ** **(95% CI)** ***n* = 104 pairs**	**ICC DZ** **(95% CI)** ***n* = 83 pairs**	**Heritability** **(95% CI)**	**Heritability *p-value***	**Unique Environmental** **Influence (95% CI)**
Fruit intake	0.14 (0.00, 0.32)	0.07 (0.00, 0.16)	0.14 (0.00, 0.32)	0.126	0.86 (0.68, 1.00)
Vegetable intake	0.39 (0.22, 0.54)	0.20 (0.11, 0.27)	0.39 (0.22, 0.54)	< 0.001	0.61 (0.46, 0.78)
Depressive symptoms	0.30 (0.13, 0.45)	0.15 (0.07, 0.22)	0.30 (0.13, 0.45)	< 0.001	0.70 (0.55, 0.87)

Heritability estimates using the AE model. *P*-values for heritability estimates were obtained by comparing the AE model with the E model.

*95% CI* 95% confidence interval, *MZ* monozygotic, *DZ* dizygotic, *ICC* intraclass correlation coefficient, Depressive symptoms based on Geriatric Depression Scale score. *P*-values for heritability under univariate AE model were obtained by comparing the log likelihoods of AE model vs E model. Fruit and vegetable intake adjusted for age, sex and total energy intake, depressive symptoms adjusted for age and sex

### Heritability analysis by sex

Intraclass correlations and heritability estimates between fruit and vegetable intake and depressive symptoms are shown in Table 4 separately for each sex (refer Supplemental Table 5 for model summary). Analysis by sex revealed that vegetable intake appeared particularly heritable in females (females 55%, males 23%). Results by sex for depressive symptoms were similar to the combined sample.

**Table 4:** Intraclass correlations and heritability estimates for fruit and vegetable intakes and depressive symptoms by sex

	**ICC MZ** **(95% CI)** ***n* = 104 pairs**	**ICC DZ** **(95% CI)** ***n* = 83 pairs**	**Heritability** **(95% CI)**	**Heritability** ***p-value***	**Unique** **Environmental** **Influence (95% CI)**
Female					
- Fruit intake	0.28 (0.13, 0.41)	0.14 (0.06, 0.20)	0.28 (0.13, 0.41)	0.012	0.72 (0.59, 0.87)
- Vegetable intake	0.55 (0.43, 0.65)	0.27 (0.22, 0.33)	0.55 (0.43, 0.65)	< 0.001	0.45 (0.35, 0.57)
- Depressive symptoms	0.30 (0.16, 0.42)	0.15 (0.08, 0.21)	0.30 (0.16, 0.42)	0.001	0.70 (0.58, 0.84)
Male					
- Fruit intake	0.04 (0.00, 0.22)	0.02 (0.00, 0.11)	0.04 (0.00, 0.22)	0.588	0.96 (0.78, 1.00)
- Vegetable intake	0.23 (0.02, 0.45)	0.11 (0.01, 0.23)	0.23 (0.02, 0.45)	0.146	0.77 (0.55, 0.98)
- Depressive symptoms	0.28 (0.06, 0.59)	0.14 (0.03, 0.30)	0.28 (0.06, 0.59)	0.050	0.72 (0.41, 0.94)

Heritability estimates using the AE model. *P*-values for heritability estimates were obtained by comparing the AE model with the E model.

*95% CI* 95% confidence interval, *MZ* monozygotic, *DZ* dizygotic, *ICC* intraclass correlation coefficient; Depressive symptoms based on Geriatric Depression Scale score. Fruit and vegetable intake adjusted for age, sex and total energy intake, depressive symptoms adjusted for age and sex

### Genetic correlations

Results for genetic correlations for depressive symptoms and (i) fruit and (ii) vegetable intakes were slightly stronger than for environmental correlations, but no significant correlations were detected (see Table 5). No significant phenotypic correlations were identified, indicating there were no significant associations between fruit and vegetable consumption and depressive symptoms.

**Table 5:** Estimates of genetic, environmental and phenotypic correlations

Traits	**Genetic correlation** **(95% CI)**	**Environmental correlation** **(95% CI)**	**Phenotypic correlation** **(95% CI)**
Fruit intake & vegetable intake	0.24 (-0.30, 0.78)	0.04 (-0.13, 0.22)	0.09 (-0.01, 0.19)
Fruit intake & depressive symptoms	-0.09 (-0.63, 0.54)	-0.05 (-0.22, 0.12)	-0.06 (-0.16, 0.04)
Vegetable intake & depressive symptoms	-0.28 (-0.65, 0.09)	0.00 (-0.17, 0.17)	-0.10 (-0.20, 0.01)

*95% CI *95% confidence interval; Depressive symptoms based on Geriatric Depression Scale score. Fruit and vegetable intake adjusted for age, sex and total energy intake, depressive symptoms adjusted for age and sex. Note: As all phenotypic correlations between fruit and vegetable intake and depressive symptoms were found not to be significant it is unlikely that genetic or environmental correlations would be significant. Analysis was exploratory in its nature

## Discussion

Our results suggest that in older adults, vegetable intake and depressive symptoms are moderately influenced by genetics (heritability 39% and 30% respectively) and that genetics has little influence on overall fruit intake. Intake of brassica vegetables appeared to be particularly heritable. We also report that shared environmental influences (such as living in the same household as children) appear not to influence fruit and vegetable intake or depressive symptoms in later life. We found no significant genetic correlation between fruit and vegetable intakes and depressive symptoms.

Most prior studies examining the heritability of dietary intake in adults have looked at participants with a wide age range and different types of diets. The exception being the paper of van der Bree et al. which examined 4640 North American adults aged ≥ 50 years and reported a 33% heritability for consumption frequency of “healthful eating habits” (comprising fruits and vegetables, dark bread, skim milk, yoghurt and rice) [20]. Studies in adult populations have reported heritability estimates ranging from 0% for fruit juice (in females 18–79 years) [16] to 54% for vegetables (in adolescents 18-19 years) [19]. Moreover, heritability of liking of fruit and vegetables was estimated to be 36% in a UK adult cohort [17]. Our results for the heritability of fruit and vegetable intake sit at the lower end of those previously reported.

Our study found differences by sex in the heritability of vegetable consumption, with an estimated heritability of 55% in women and 23% in men. Two previous studies have also reported sex differences in heritability of vegetable intake [15, 18]. These studies examined younger cohorts (Danish twins aged ≥ 18 years [15] and Finnish twins aged 22–27 years [18]) than the current study making it difficult to draw comparisons. However, it appears heritability of vegetable intake may vary by sex.

The current study found the heritability of depressive symptoms to be 30%, with similar results in both females and males. This is supported by results previously reported in older adults (heritability estimates ranged from 18% to 55% [41–44]).

The current study’s finding that shared environmental factors do not influence intake of fruit and vegetables in older adults’ is not unexpected given the results of other studies on the heritability of diet [15, 18–20, 45]. It has been demonstrated that the shared environment influences children’s preferences for fruit and vegetables, with a study of 2686 UK 3-year-old children reporting a 35% contribution for both fruit and vegetables [46]. However, it appears that once a person reaches adulthood, the influence of the shared environment decreases [45]. For example, a study of 4388 Finnish twins aged 22 to 27 years found no influence of the shared environment when examining the heritability of dietary intake, which included the intake of vegetables, fruits and berries [18]. The lack of influence of the shared environment on an older adults’ dietary intake suggests that targeting changes to the food environment over the lifespan is likely to be more effective at changing consumption in older adults than only targeting changes in childhood environments.

Several mechanisms, both physiological and psychological, may explain the relationship detected between genetics and vegetable intake. Taste perception appears to have a genetic component, for example variation in the bitter taste receptor gene *TAS2R38* has been associated with vegetable preferences [47]. Lower genetic sensitivity to bitter taste has been associated with higher bitter tasting vegetable consumption [48]. Our study’s finding that brassica vegetables had the highest heritability supports this previous research as the bitterness of brassica vegetables has been shown to be the least tolerated vegetable group [49]. Inherited personality traits may also contribute to the heritability of vegetable intake with eating behaviours, including emotional eating [50] and “adventurous” eating [51] appearing to have a genetic component.

Our results provide potential insights into drivers of fruit and vegetable consumption in older adults. It appears that fruit intake may be particularly influenced by changes in the environment as our results suggest genetics has little influence on fruit intake. The finding that vegetable intakes are particularly heritable in women, suggests that changes in the environment may have less impact on vegetable consumption in women than men. However, this result should be viewed with caution due to the low number of male participants. Environmental changes may have less impact on intakes of bitter tasting brassica vegetables than interventions targeting consumption of root vegetables (e.g. carrots). However, there is the possibility of increasing the consumption of bitter tasting vegetables by breeding varieties with less bitter taste or by communicating preparation instructions to mask this taste [49]. Further research is required to identify genes associated with the consumption of different vegetables. The output of which could be used to develop individualised interventions aimed to increase vegetable intake based on a person’s genetic predisposition to consuming different vegetables.

Our exploratory analysis into whether there are shared genetic influences on fruit and vegetable intake and depressive symptoms found no significant associations. This is unsurprising given we also report no phenotypic association in our cohort. A beneficial cross-sectional association has previously been reported between fruit and vegetable intakes and depression in studies of older adults [52, 53]. However, these studies contained participants with higher levels of depression than in our twin cohort (20% and 52% versus 7% in our cohort). It is unclear if our finding of no genetic correlation between fruit and vegetable intake and depressive symptoms is due to our study’s sample size and our participants’ relatively low overall level of depressive symptoms (which has reduced the variability in depression scores) or if there is no underlying correlation.

Limitations of the current study should be considered when interpreting results. Firstly, this study included a modest sample size which did not allow us to perform a full analysis of differences by sex. Secondly, like most dietary collection tools, FFQs are subject to inaccuracies. However, FFQs provide useful data in comparing individuals according to their food intakes [54], which is of importance to this study. Total energy intake was also included in the model to correct for over or underreporting of total intake. Nevertheless, some inaccuracies are still likely to exist and may increase measurement error and hence decrease heritability estimates. Thirdly, a large number of participants were excluded from the final sample used for analysis due to not providing dietary data or not having their co-twin provide data at the same time point. This may have limited our results. Fourthly, our sample included well educated, white older Australians. Eating habits are likely to be specific to this group and therefore our results may not be generalisable to other populations.

There are a number of strengths of this study, including its narrow age range for participants. Heritability appears to change across the lifespan, with little known of the heritability of diet in older adults. Our study helps address this gap in the literature. Also, by using an Australian cohort we have broadened the geographical area examined in studies on heritability of diet as prior work has been restricted to the UK, US and Northern Europe.

## Conclusions

Our findings provide novel insights into the drivers of fruit and vegetable intakes in older adults. Consumption of vegetables, particularly bitter tasting brassica vegetables, was significantly influenced by genetics, with environmental influences also apparent. The total consumption of fruit was only influenced by the environment, with no genetic influence detected, suggesting strategies targeting the food environment may be particularly effective for encouraging fruit consumption. Further research is required to confirm these results and to fully examine differences by sex. We report that the childhood environment shared by twins did not influence their consumption of fruit and vegetables as older adults, which suggests strategies targeting the whole lifespan are likely to be more effective than strategies only targeting the childhood food environment. Our exploratory analysis found no genetic correlation between fruit or vegetable intakes and depression.

## Supplementary Information

**Additional file 1: Supplemental Table 1.** Heritability Analysis: Heritability estimates for intakes of individual types of fruit and vegetables. **Supplemental Table 2.** Goodness of fit for AE, CE and E model compared with the full ACE model. **Supplemental Table 3.** Sensitivity Analysis: Heritability estimates for intakes of individual types of fruit and vegetables adjusted for education and BMI. **Supplemental Table 4.** Sensitivity analysis: Goodness of fit for AE, CE and E model compared with the full ACE model. **Supplemental Table 5.** Tri-variate Model summary.

## Data Availability

Data supporting this study are available upon request with the corresponding author, Annabel Matison. Data from the Older Australian Twins Study (https://cheba.unsw.edu.au/research-projects/older-australian-twins-study) may be requested by contacting the CHeBA Research Bank at CHeBAData@unsw.edu.au.

## Abbreviations

A: Additive genetic influences
AIC: Akaike information criteria
BMI: Body mass index
C: Shared environmental factors
CI: Confidence interval
DQESv2: Dietary Questionnaire for Epidemiological Studies Version 2
DZ: Dizygotic
DZICC: Dizygotic twin intraclass correlation coefficient
E: Unique environmental influences
FFQ: Food frequency questionnaire
GDS: Geriatric Depression Scale
MZ: Monozygotic
MZICC: Monozygotic twin intraclass correlation coefficient
OATS: Older Australian Twins Study
SD: Standard deviation
SEM: Structural equation modelling
