# Supplementary material for: Genetic and environmental influences on fruit and vegetable consumption and depression in older adults
Source: BMC Geriatr. 2023 Feb 3;23:67. doi: 10.1186/s12877-023-03745-0 (PMC9896753; doi:10.1186/s12877-023-03745-0)
Supplement: Supplementary file 1 — Additional file 1: Supplemental Table 1. Heritability Analysis: Heritability estimates for intakes of individual types of fruit and vegetables. Supplemental Table 2. Goodness of fit for AE, CE and E model compared with the full ACE model. Supplemental Table 3. Sensitivity Analysis: Heritability estimates for intakes of individual types of fruit and vegetables adjusted for education and BMI. Supplemental Table 4. Sensitivity analysis: Goodness of fit for AE, CE and E model compared with the full ACE model. Supplemental Table 5. Tri-variate Model summary. [file 12877_2023_3745_MOESM1_ESM.docx]

**Supplemental Table 1 Heritability Analysis:** Heritability estimates for intakes of individual types of fruit and vegetables

| **Type of fruit/vegetable** | **Heritability (genetic**  **influence)**  **(95% CI)** | ***p-value*** | **Unique environmental influence**  **(95% CI)** | **ICC MZ**  **(95% CI)** | **ICC DZ**  **(95% CI)** |
| --- | --- | --- | --- | --- | --- |
| **Tropical/citrus fruit** | 0.22 (0.04, 0.38) | 0.019 | 0.78 (0.62, 0.96) | 0.22 (0.04, 0.38) | 0.11 (0.02, 0.19) |
| **Stone fruit** | 0.18 (0.0, 0.36) | 0.060 | 0.82 (0.64, 1.0) | 0.18 (0.0, 0.36) | 0.09 (0.0, 0.18) |
| **Other fruit** | 0.16 (0.0, 0.35) | 0.100 | 0.84 (0.65, 1.0) | 0.16 (0.0, 0.35) | 0.08 (0.0, 0.17) |
| **Brassica vegetables** | 0.40 (0.24, 0.54) | <0.001 | 0.60 (0.46, 0.76) | 0.40 (0.24, 0.54) | 0.20 (0.12, 0.27) |
| **Root vegetables** | 0.17 (0.0, 0.36) | 0.083 | 0.83 (0.64, 1.0) | 0.17 (0.0, 0.36) | 0.09 (0.0, 0.18) |
| **Salad vegetables** | 0.26 (0.08, 0.42) | 0.005 | 0.74 (0.58, 0.92) | 0.26 (0.08, 0.42) | 0.13 (0.04, 0.21) |
| **Starchy vegetables** | 0.20 (0.02, 0.36) | 0.029 | 0.80 (0.64, 0.98) | 0.20 (0.02, 0.36) | 0.10 (0.01, 0.18) |
| **Peas/beans/legumes** | 0.28 (0.12, 0.42) | 0.001 | 0.72 (0.58, 0.88) | 0.28 (0.12, 0.42) | 0.14 (0.06, 0.21) |
| **Other vegetables** | 0.20 (0.03, 0.35) | 0.020 | 0.80 (0.65, 0.97) | 0.20 (0.03, 0.35) | 0.10 (0.02, 0.17) |

Heritability estimates under the univariate AE model. P-values for heritability estimates were obtained by comparing the AE model with the E model. All intakes adjusted for age, sex and total energy intake. 95% CI – 95% confidence interval; MZ – monozygotic, DZ – dizygotic; ICC intraclass correlation coefficient

**Supplemental Table 2** Goodness of fit for AE, CE and E model compared with the full ACE model

| **Trait** | **AIC.ACE** | **AIC.AE** | **AIC.CE** | **AIC.E** | **AE *p-value*** | **CE *p-value*** | **E *p-value*** |
| --- | --- | --- | --- | --- | --- | --- | --- |
| **Fruit** | 319.31 | 317.31 | 318.61 | 317.64 | 1.000 | 0.253 | 0.311 |
| **Vegetables** | 303.27 | 301.27 | 304.20 | 317.90 | 1.000 | 0.087 | <0.001 |
| **Depressive symptoms** | 308.94 | 307.08 | 307.48 | 317.18 | 0.707 | 0.463 | 0.002 |
| **Tropical/citrus fruit** | 315.85 | 313.85 | 315.10 | 317.36 | 1.000 | 0.264 | 0.064 |
| **Stone fruit** | 318.03 | 316.03 | 318.14 | 317.57 | 1.000 | 0.147 | 0.170 |
| **Other fruit** | 319.82 | 317.82 | 319.74 | 318.53 | 1.000 | 0.166 | 0.259 |
| **Brassica vegetables** | 300.19 | 298.70 | 299.09 | 317.51 | 0.476 | 0.341 | <0.001 |
| **Root vegetables** | 320.02 | 318.02 | 320.25 | 319.03 | 1.000 | 0.135 | 0.222 |
| **Salad vegetables** | 312.69 | 311.12 | 310.79 | 316.95 | 0.513 | 0.752 | 0.016 |
| **Starchy vegetables** | 317.17 | 315.63 | 315.18 | 318.40 | 0.497 | 0.954 | 0.073 |
| **Peas/beans/legumes** | 310.27 | 308.27 | 310.02 | 317.87 | 1.000 | 0.186 | 0.003 |
| **Other vegetables** | 314.96 | 314.57 | 312.96 | 317.99 | 0.204 | 1.000 | 0.030 |

A - genetic influence; C - shared environmental influence; E - unique environmental influence; AIC – Akaike information criterion (lower values reflect better model fit)

**Supplemental Table 3 Sensitivity Analysis:** Heritability estimates for intakes of individual types of fruit and vegetables adjusted for education and BMI

| **Type of fruit/vegetable** | **Heritability (genetic**  **influence)**  **(95% CI)** | ***p-value*** | **Unique environmental influence**  **(95% CI)** | **ICC MZ**  **(95% CI)** | **ICC DZ**  **(95% CI)** |
| --- | --- | --- | --- | --- | --- |
| **Fruit intake** | 0.13 (0.0, 0.31) | 0.163 | 0.87 (0.69, 1.0) | 0.13 (0.0, 0.31) | 0.07 (0.0, 0.15) |
| **Vegetable intake** | 0.39 (0.22. 0.53) | <0.001 | 0.61 (0.47, 0.78) | 0.39 (0.22, 0.53) | 0.19 (0.11, 0.27) |
| **Depressive symptoms** | 0.29 (0.12, 0.43) | <0.001 | 0.71 (0.57, 0.88) | 0.29 (0.12, 0.43) | 0.14 (0.06, 0.22) |
| **Tropical/citrus fruit** | 0.22 (0.04, 0.38) | 0.018 | 0.78 (0.62, 0.96) | 0.22 (0.04, 0.38) | 0.11 (0.02, 0.19) |
| **Stone fruit** | 0.18 (0.0, 0.36) | 0.063 | 0.82 (0.64, 1.0) | 0.18 (0.0, 0.36) | 0.09 (0.0, 0.18) |
| **Other fruit** | 0.16 (0.0, 0.34) | 0.112 | 0.84 (0.66, 1.0) | 0.16 (0.0, 0.34) | 0.08 (0.0, 0.17) |
| **Brassica vegetables** | 0.38 (0.22, 0.52) | <0.001 | 0.62 (0.48, 0.78) | 0.38 (0.22, 0.52) | 0.19 (0.11, 0.26) |
| **Root vegetables** | 0.17 (0.0, 0.36) | 0.096 | 0.83 (0.64, 1.0) | 0.17 (0.0, 0.36) | 0.08 (0.0, 0.18) |
| **Salad vegetables** | 0.25 (0.07, 0.41) | 0.007 | 0.75 (0.59, 0.93) | 0.25 (0.07, 0.41) | 0.12 (0.03, 0.20) |
| **Starchy vegetables** | 0.18 (0.0, 0.35) | 0.045 | 0.82 (0.65, 1.0) | 0.18 (0.0, 0.35) | 0.09 (0.00, 0.17) |
| **Peas/beans/legumes** | 0.27 (0.11, 0.41) | 0.001 | 0.73 (0.59, 0.89) | 0.27 (0.11, 0.41) | 0.13 (0.05, 0.21) |
| **Other vegetables** | 0.18 (0.01, 0.33) | 0.035 | 0.82 (0.67, 0.99) | 0.18 (0.01, 0.33) | 0.09 (0.01, 0.17) |

Heritability estimates under the univariate AE model. P-values for heritability estimates were obtained by comparing the AE model with the E model. All intakes adjusted for age, sex, education, BMI and total energy intake, depressive symptoms adjusted for age, sex, education and BMI. 95% CI – 95% confidence interval; MZ – monozygotic, DZ – dizygotic; ICC intraclass correlation coefficient

**Supplemental Table 4** Sensitivity analysis: Goodness of fit for AE, CE and E model compared with the full ACE model

| **Trait** | **AIC.ACE** | **AIC.AE** | **AIC.CE** | **AIC.E** | **AE *p-value*** | **CE *p-value*** | **E *p-value*** |
| --- | --- | --- | --- | --- | --- | --- | --- |
| **Fruit** | 319.51 | 317.51 | 318.79 | 317.46 | 1.000 | 0.259 | 0.378 |
| **Vegetables** | 303.84 | 301.84 | 304.64 | 318.01 | 1.000 | 0.094 | <0.001 |
| **Depressive symptoms** | 309.88 | 308.08 | 308.24 | 317.07 | 0.649 | 0.544 | 0.004 |
| **Tropical/citrus fruit** | 316.17 | 314.17 | 315.51 | 317.75 | 1.000 | 0.248 | 0.062 |
| **Stone fruit** | 318.22 | 316.22 | 318.31 | 317.68 | 1.000 | 0.148 | 0.177 |
| **Other fruit** | 320.17 | 318.17 | 320.10 | 318.70 | 1.000 | 0.165 | 0.282 |
| **Brassica vegetables** | 302.42 | 301.32 | 300.78 | 317.61 | 0.344 | 0.548 | <0.001 |
| **Root vegetables** | 320.94 | 318.94 | 321.07 | 319.72 | 1.000 | 0.145 | 0.249 |
| **Salad vegetables** | 313.32 | 311.76 | 311.40 | 317.04 | 0.506 | 0.786 | 0.021 |
| **Starchy vegetables** | 318.50 | 316.90 | 316.50 | 318.93 | 0.526 | 0.970 | 0.109 |
| **Peas/beans/legumes** | 311.45 | 309.45 | 311.40 | 317.81 | 1.000 | 0.163 | 0.006 |
| **Other vegetables** | 316.53 | 316.03 | 314.53 | 318.49 | 0.220 | 1.000 | 0.051 |

A - genetic influence; C - shared environmental influence; E - unique environmental influence; AIC – Akaike information criterion (lower values reflect better model fit)

**Supplemental Table 5** Tri-variate Model summary

| **Name** | **Ep** | **-2LL** | **AIC** | **diffLL** | **Diffdf** | ***p-value*** |
| --- | --- | --- | --- | --- | --- | --- |
| **CholACE** | 21 | 3141.03 | 939.03 | - | - | - |
| **CholAE** | 15 | 3143.96 | 929.96 | 2.93 | 6 | 0.817 |

Ep - estimated parameters; -2LL - negative of twice of log likelihood; AIC – Akaike information criterion; diffLL difference in log likelihoods when compared with ACE model; diffdf – difference in degrees of freedom when compared to ACE model. *P-value* – Comparison of likelihoods of ACE vs AE.
